# Emerging Roles for Ion Channels in Ovarian Cancer: Pathomechanisms and Pharmacological Treatment

**DOI:** 10.3390/cancers13040668

**Published:** 2021-02-07

**Authors:** Concetta Altamura, Maria Raffaella Greco, Maria Rosaria Carratù, Rosa Angela Cardone, Jean-François Desaphy

**Affiliations:** 1 Department of Biomedical Sciences and Human Oncology, School of Medicine, University of Bari Aldo Moro, 70124 Bari, Italy; grecoraffaella@hotmail.it (M.R.G.); mariarosaria.carratu@uniba.it (M.R.C.); jeanfrancois.desaphy@uniba.it (J.-F.D.); 2 Department of Biosciences, Biotechnologies, and Biopharmaceutics, University of Bari Aldo Moro, 70125 Bari, Italy; rosaangela.cardone@uniba.it

**Keywords:** ion channels, ovarian cancer, drug resistance, chemoresistance

## Abstract

**Simple Summary:**

Around 4% of cancer deaths in the world was associated to ovarian cancer (OC), making it the eighth most common cause of death in women. Increasing evidence suggests that ion channels play critical role in the main stages of OC process, including proliferation, migration and metastasis. The aim of this review was to provide an updated description of the current knowledge concerning ion channels’ involvement in OC, with a particular emphasis of their role in the acquired chemoresistance. Importantly, ion channels might represent new molecular targets for the development of OC treatment, exploiting the availability of the well-known ion channel-targeting drugs.

**Abstract:**

Ovarian cancer (OC) is the deadliest gynecologic cancer, due to late diagnosis, development of platinum resistance, and inadequate alternative therapy. It has been demonstrated that membrane ion channels play important roles in cancer processes, including cell proliferation, apoptosis, motility, and invasion. Here, we review the contribution of ion channels in the development and progression of OC, evaluating their potential in clinical management. Increased expression of voltage-gated and epithelial sodium channels has been detected in OC cells and tissues and shown to be involved in cancer proliferation and invasion. Potassium and calcium channels have been found to play a critical role in the control of cell cycle and in the resistance to apoptosis, promoting tumor growth and recurrence. Overexpression of chloride and transient receptor potential channels was found both in vitro and in vivo, supporting their contribution to OC. Furthermore, ion channels have been shown to influence the sensitivity of OC cells to neoplastic drugs, suggesting a critical role in chemotherapy resistance. The study of ion channels expression and function in OC can improve our understanding of pathophysiology and pave the way for identifying ion channels as potential targets for tumor diagnosis and treatment.

## 1. Introduction

Ovarian cancer (OC), nicknamed “the silent killer,” is one of the most dangerous cancers for women. Annually, OC accounts for 3.6% of all cancer new cases (about 239,000) and 4.3% of all cancer deaths (about 152,000) worldwide. This makes OC the eighth most common cause of cancer death in women and the second most common cause of gynecologic cancer death after cancer of the cervix uteri [1,2].

The underlying mechanism that leads to OC is not well understood but it is likely related to the processes of reproduction and ovulation. A number of factors may increase the risk of developing OC such as nulliparity, infertility, endometriosis, obesity, and late age. Conversely, gravidity, breastfeeding, and oral contraceptive use may reduce this risk. In addition, epidemiologic evidences suggest a lower risk of developing OC for women regularly using nonsteroidal anti-inflammatory agents, including aspirin [3].

About 10% of OCs are linked to genetic predisposition [4]. Most common are the mutations in the tumor suppressor genes *BRCA1* and *BRCA2* (Breast Cancer related Antigen), which can increase the risk of OC from 1.6% to 40% and 18%, respectively [5]. 

Although cancers classed as “ovarian” are remarkably heterogeneous, more than 90% of them originate from the ovarian mesothelium [6]. Malignant cells may spread to the peritoneal fluid where they form aggregates able to implant in or onto the peritoneal cavity wall or invade other pelvic organs [7]. In the early stage, the disease is characterized by few and unspecific symptoms such as abdominal pain, swelling, or nonspecific gastrointestinal symptoms, which can be confused with other nonmalignant conditions [8].

This means that the majority of women are not diagnosed until the disease has reached an advanced stage, associated with a poor diagnosis, when the tumor has spread to the upper abdomen, reaching lymphatic vessels and brain. Thus, early diagnosis and effective therapy are of utmost importance in order to improve survival rates. 

The cancer antigen 125 (CA125), historically used as a biomarker in the early diagnosis of OC, is no longer recommended for OC screening because it lacks in specificity and sensitivity. However, the combination of CA125 with the Human Epididymis protein 4 (HE4) and the transvaginal sonography could be effective to detect OC early and to estimate potential risk factors [9].

Regarding therapy, primary debulking surgery followed by platinum-taxane therapy has represented the standard of care in the treatment of OC for more than four decades [10], but relapse often occurs within a few months [11]. In the past decade, well-conducted clinical trials have allowed the definition of different lines of targeted therapy for patients with OC. The introduction of concurrent bevacizumab and sequential bevacizumab and PARP inhibitors have improved progression-free survival and less toxicity in some OC patients, who developed recurrence within the first six months of front line platinum and taxane therapies [2]. 

Despite the positive impact of these drugs in OC treatment, there is a need for therapies that provide longer disease-free survival, especially for patients whose cancers are platinum-resistant or platinum-refractory. Therefore, the discovery of targets with important functions in OC progression and prognosis might facilitate the development of novel therapeutic strategies. In this line, recent studies have demonstrated the contribution of ion channels in the progression of various tumors, including breast cancer, prostate cancer, and colon cancer [12]. It has been established that ion channels influence a variety of cellular processes, many of which are essential for maintaining tissue homeostasis such as cell proliferation, migration, and apoptosis. Alterations in channels’ expression and/or function can induce the transformation of normal cells into malignant ones. The latter show uncontrolled multiplication and spreading, which are the hallmarks of cancer [13]. The term oncochannelopathy was coined to define this tight association between cancer and ion channel dysfunction [14].

Growing evidence has suggested that ion channel expression/activity could also play a role in the pathophysiology of OC and could represent new targets for treatment [15]. Ion channels are pore-forming membrane proteins found in all human tissues, whose opening allows the passive flow of selected ions following their electrochemical gradient. Ion redistribution between the extracellular and intracellular environments profoundly changes the electrochemical properties of the cells and modulates intracellular signaling and enzymes, thereby affecting cell function. Therefore, ion channels play a pivotal role in a variety of biological processes such as the flow of nerve impulses, muscle contraction, signal transduction, endocrine and exocrine secretion, cell growth, migration, apoptosis, and differentiation [16,17,18]. Consistent with the distribution and the roles of ion channels throughout the human body, defects in ion channel expression and/or activity have been implicated in a wide variety of diseases. For instance, ion channels are involved in diabetes, hypertension, cardiac arrhythmias and cardiomyopathy, neurologic and neuromuscular disorders, pain and cancer [19,20,21].

The goal of this review is to summarize current knowledge about the role of ion channel dysfunction in cancer development in the ovary. We point out the potential of these ion channel proteins to serve as potential targets for tumor diagnosis and treatment. Although the mechanisms accounting for the impact of ion channel modulators on cancer have not been fully clarified, numerous in vivo and in vitro experiments targeting these proteins argue for the feasibility of applying ion channels modulators in cancer therapy. Yet, it should be kept in mind that many of these compounds might involve other targets and mechanisms of action (Table 1). Notably, some data also suggest the possible involvement of ion channels in the mechanisms of resistance to chemotherapy in OC.

## 2. Sodium Channels

Two major classes of sodium channels are expressed in mammals: the voltage-gated sodium channels (VGSC) and the epithelial sodium channels (eNaC). Nine isoforms (Nav1.1–Nav1.9) of VGSC are expressed throughout the body in various excitable cell types, while epithelial sodium channels are found mainly in the skin, lung, and kidney [22]. Altered expression of these channels has been observed in various tumors, especially in breast cancer, colon cancer, prostate cancer, and small cell lung cancer [23,24,25,26,27].

The first direct hints that VGSCs may be important for ovarian carcinogenesis came in 2010, when Gao and collaborators investigated the functional expression of these channels in OC and the possible correlation between VGSC activity and carcinogenic processes [28]. Gene expression analysis demonstrated that mRNA levels of Nav1.1, Nav1.3, Nav1.4, and Nav1.5 are significantly higher in OC cells than those in normal ovarian tissue, suggesting a role of VGSC in ovarian tumorigenesis. Furthermore, Nav1.2, Nav1.4, Nav1.5, and Nav1.7 mRNA levels are greatly increased in the highly metastatic OC cell lines compared to the low-metastatic cells, correlating the enhanced expression of VGSC with the cellular invasion process. Additional experiments revealed that the Nav1.5 protein is the main VGSC involved in OC and that increased Nav1.5 expression was associated with high histological grade and metastasis in OC [28].

**Figure 1:**
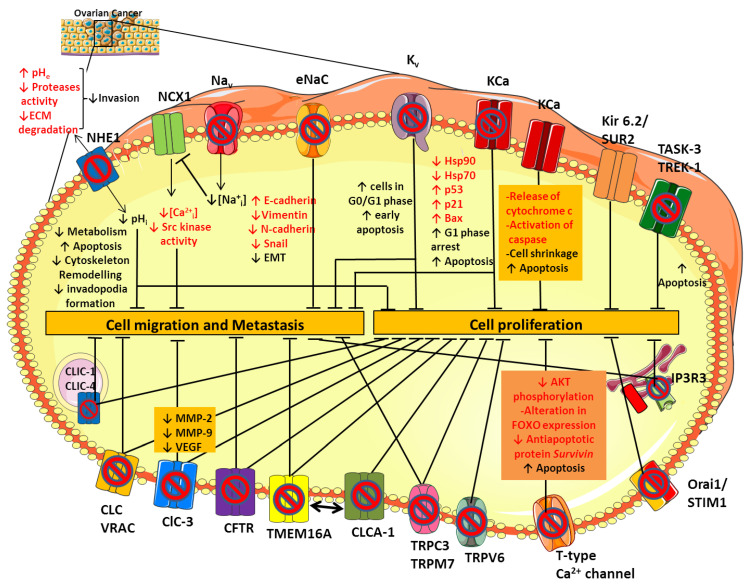
Ion channels expression in ovarian cancer (OC). Schematic representation of the main ion channels and transporters involved in OC development and progression. Their dysregulated role in cell proliferation, migration and metastasis has been studied by modulating their activity with selective ion channel inhibitors. See text for further details.

To date, the molecular pathway by which sodium channels contribute to cancer progression and invasiveness is not fully understood yet, but there are some hypotheses. The VGSCs expressed in cancer cells, including Nav1.5, carry a small persistent Na^+^ current that depolarizes the resting potential and induces cytosolic Na^+^ accumulation (Figure 1) [29,30]. Elevated intracellular Na^+^ leads to Ca^2+^ entry through the reverse mode activity of the sodium-calcium exchanger (NCX1) [31], thus triggering activation of Src kinases that promote cancer cell invasion and metastatic progression (Figure 1) [32]. The NCX1-mediated increase in Na^+^ efflux may activate the isoform 1 of the sodium-proton exchanger NHE1 [31] consequently leading to intracellular alkalinisation and extracellular acidification. These conditions stimulate tumor cell metabolism, promote the formation and the activity of actin-rich invasive protrusions, the invadopodia, and activates acidic-pH-dependent proteases such as the zinc-dependent matrix metalloproteases MMP-2 and -9 [33] and/or the acidic cysteine proteases [34], leading to an increase in proteolysis-dependent cell invasion (Figure 1) [35]. In line with this, inhibition of NHE1 activity with selective inhibitors, such as Cariporide and Zoniporide reduces cancer cell proliferation and invasive capability in different tumor types, including OC (Figure 1) [36,37]. Moreover, it has been shown that both in CHO cells and in the human OC cells A2780 NHE1 and NCX1 are physically and functionally linked such that an increase in sodium concentration caused by NHE1 can further drive NCX1 to generate a coordinate sodium efflux in exchange for calcium entry [31,38]. Thus, the resulting calcium increase inside the cell can promote tumorigenesis and tumor progression [39] (Figure 1).

Because the functional activity of VGSC is required to promote OC cell invasiveness, they constitute a promising target for the OC drug discovery. It was demonstrated that the treatment of OC cell lines (SKOV-3 and Caov-3) with the selective VGSD blocker tetrodotoxin (TTX) (Table 1) reduces the migration and invasion by 50–60%, without any significant effect on proliferation [28]. However, this natural agent is unequivocally toxic for mammals.

Recently, several population-based studies reported that polyunsaturated fatty acids, especially the omega 3 eicosapentaenoic acid (EPA) (Table 1), have a protective effect against OC [40,41]. Treatment of OC cells with EPA is associated with suppression of growth and reduction in invasivity [42]. It appeared that EPA exerts these beneficial effects through inhibition of Nav1.5 channels in a dose-dependent manner [43]. Additionally, various VGSC blockers belonging to class 1B antiarrhythmic drugs, antiepileptic drugs, and local anesthetics have been studied in in vitro and in vivo cancer models [44]. For instance, treatment of OC cells with the antiepileptic topiramate reduced OC cell proliferation and migration, suggesting a potential anticancer effect (Table 1) [45]. As regards the local anesthetics, retrospective studies have revealed that the use of these drugs for regional anesthesia during perioperative period reduced the recurrence rate of patients with breast cancer [46]. In keeping with this, in vitro experiments demonstrated that bupivacaine and lidocaine inhibited OC cells proliferation and metastasis by blocking Nav1.5, confirming their role as potential anticancer agents [47,48]. The multi-effects of lidocaine were also confirmed in vivo, by using murine syngeneic OC (ID8) model. The molecular mechanism by which lidocaine hampers OC malignancy was recently studied by Liu and collaborators, who demonstrated that lidocaine binding to Nav1.5 blocked the activation of FAK/Paxillin signaling pathway, inhibiting proliferation and metastasis, and decreased cisplatin resistance of OC cells [48]. However, it should be noted that these compounds might elicit their anticancer effects also through mechanisms other than VGSC inhibition. Indeed, Xuan et al. demonstrated that bupivacaine showed antiproliferative effects via the direct activation of intrinsic and extrinsic apoptotic pathways [47].

There has also been interest in developing ion channel-targeting antibodies. E3Ab, a polyclonal antibody recognizing the third extracellular region of Nav1.5, inhibits approximately 60% of sodium currents in HEK-293 and CHO cells, without affecting Nav1.4 or Nav1.6 isoforms (Table 1) [49]. The antibody was shown to inhibit tumor growth and invasive capacity of OC cells, indicating the therapeutic potential of E3Ab in Nav1.5-positive OC [50].

**Table 1:** Ion channels blockers and openers tested in OC and their biological effects.

Drug	Clinical Indications	Ion Channel Target	Biological Effects in OC	Other Known Effects/Uses	Ref.
TTX	Natural toxin	VGSC blocker	In vitro: reduction of OC cells migration and invasion	-	[28]
EPA	Omega-3 fatty acids	Nav1.5 blocker	In vitro: suppression of growth and reduction of invasivity	Cardioprotective effects, cognitive function improvement, fatigue recovery and endurance performance improvement, maintenance of immune function	[42,43,51]
Topiramate	Antiepileptic drug	VGSC blocker	In vitro: reduction of cell proliferation and migration	Off-label uses: neuropathic pain, psychotropic drug-induced weight gain, alcohol use disorders with tobacco dependence, binge eating disorder, bulimia nervosa, obesity with hypertension, adjunctive therapy in bipolar disorder, unipolar depression, borderline personality disorder, obsessive-compulsive disorder, posttraumatic stress disorder, Tourette syndrome, Prader-Willie syndrome, essential tremor. Carbonic anhydrase inhibitor	[45,52]
Bupivacaine	Local anesthetic drug	VGSC blocker	In vitro: reduction of OC cell proliferation and migration	-	[47]
Lidocaine	Local anesthetic drug	VGSC blocker	In vitro: reduction of OC cell proliferation, migration, and invasion; increase of cisplatin efficiency in OC cells In vivo: increase of cisplatin efficiency in a murine ovarian metastatic model	Intractable cough and asthma, and chronic (including neuropathic) pain.	[48,53,54]
E3Ab	Polyclonal antibody	Nav1.5	In vitro: reduction of OC cell proliferation and invasive capacity	-	[49,50]
Imipramine	Tricyclic antidepressant drug	Eag blocker	In vitro: early apoptosis	Inhibitor of the reuptake of norepinephrine and serotonin; Antagonist of D2 dopamine, muscarinic, α1 and α2 adrenergic, and H1 histamine receptors. Off-label uses: chronic neuropathic pain and panic disorder.	[18,55,56,57]
Clofilium	Antiarrhythmic drug	Eag blocker	In vitro: early apoptosis	In mammalian cardiac myocytes, clofilium also affects Na^+^ and L-type Ca^2+^ currents. NMDA glutamate Rc antagonist.	[55,58,59]
Berberine	Natural alkaloid	hERG blocker	In vitro: inhibition of OC cells proliferation	Antiviral activity against Herpes simplex virus 1 and 2 by modulating host cell activation of the NF-kB and MAPK pathway.	[60,61]
mAb56	Monoclonal antibody	Eag blocker	In vitro: inhibition of OC cell growth	-	[62]
Tetrandrine	Natural alkaloid	BK channels blocker	In vitro: reduction of OC cell proliferation by inducing G1 phase arrest and accelerating apoptosis	Anti-hypertensive effects through T-type Ca^2+^ channel blocking; Anti-inflammatory, immunosuppressant and antiallergic effects mediated by tetrandrine inhibition of ILs, TNF-a, prostaglandin, cycloxygenase-2 and T cells; Antioxidant activity by scavenging the superoxide anion radicals; Anticancer agent; Antimicrobial activity.	[63,64]
Iberiotoxin	Natural toxin	BK channels blocker	In vitro: reduction of OC cell proliferation by inducing G1 phase arrest	-	[63]
NS1619	Experimental compound	BK channels opener	In vitro: inhibition of OC cell proliferation by cell shrinkage and release of cytochrome c and activation of caspase, inducing apoptosis	Protection of cardiac muscle against ischaemia and reperfusion injury; Cytoprotective effect through directly inhibition of SERCA activity.	[65,66]
Minoxidil	Antihypertensive drug	Kir6.2/SUR2 activator	In vitro: reduction of OC cell proliferation In vivo: Reduction of tumor growth in OC xenograft model	Treatment for androgenetic alopecia and off-label uses for other hair loss conditions	[67,68]
Methanandamide	Cannabinoid receptor agonist	TASK-3 blocker	In vitro: reduction of OC cell proliferation and increasing in apoptosis	Anti- and pronociceptive effects by activating both cannabinoid (CB1) and vanilloid (TRPV1) receptors of nociceptive primary afferents.	[69,70]
Zinc	Chemical element	TASK-3 blocker	In vitro: reduction of OC cells proliferation and increase of apoptosis	Anti-diarrheal effects by inhibiting cyclic adenosine monophosphate (cAMP), calcium, and nitric oxide; antioxidative and antimicrobial effects through the antioxidant activity of cysteine-rich metallothioneins	[69,71]
Curcumin	Natural phenolic antioxidant	TREK-1 blocker	In vitro: reduction of OC cells proliferation and increase of late apoptosis processes	Antioxidant, anti-inflammatory, antimicrobial, antidiabetic, anti-aging effects. Multi-ion channel blocker such as voltage-gated Na^+^, Ca^2+^, K^+^ channels Activation of various TRP channels.	[72,73,74,75]
Amphotericin	Antifungal	K^+^ ionophore	In vitro: sensitize cells to cisplatin and other platinum-based agents	Production of free radicals and subsequently oxidative damage; stimulation of phagocytic cells	[76,77]
Bumetanide	Diuretic	Na-K-Cl cotransporter inhibitor	In vitro: boost cisplatin-induced apoptosis	-	[78]
Niflumic acid	Nonsteroidal anti-inflammatory drug	CLC and VRAC blocker	In vitro: inhibition of OC cell proliferation, adhesion, and invasion	Blocking effect on TRPV1 channels, T-type calcium channels, CFTR, TRPA1, and several voltage-gated potassium channels.	[79,80,81,82,83,84]
		CLCA1 blocker	In vitro: Reduced ability of OC cells to form multicellular aggregates		[85]
Tamoxifen	Non-steroidal antiestrogen	CLC and VRAC blocker	In vitro: Inhibition of OC cell proliferation and suppression of cell adhesion and invasion	Treatment of hormone-sensitive breast cancer	[79]
Nitro-2-(3-phenylpropylamino)-benzoate (NPPB)	Experimental compound	CLC and VRAC blocker	In vitro: Inhibition of OC cell proliferation and suppression of cell adhesion and invasion	-	[80,86]
Mibefradil	In a phase I clinical trial for the treatment of gliomas	T-type Ca^2+^ channel blocker	In vitro: Alteration of cell-cycle progression, slowing of proliferation, and increase of cell death processes Inhibition of growth and increase of apoptosis in platinum-resistant OC cells In vivo: mibefradil sensitizes platinum-resistant OC to carboplatin in a mouse model of peritoneal metastasis due to OC	Blocking effects on Orai channels (204 Li et al., 2019); Inhibition of CYP3A cytochrome (205 Foti et al., 2011); inhibition of Kv10.1 (206 Gómez-Lagunas et al., 2017) In a phase I clinical trial for the treatment of gliomas	[87,88,89,90,91,92]
Nifedipine	antihypertensive and antianginal	Voltage-gated Ca^2+^ channel blocker	In vitro: Inhibition of LPA-induced OC cell migration and adhesion; Increase of cytosolic calcium levels in A2780 cells	Off-label uses: Raynaud phenomenon; Tocolysis; Distal ureteric calculi	[93,94,95,96]
Sulforaphane (SFN)	Natural isothiocyanate compound	IP3R1	In vitro: Inhibition of OC cell proliferation	Antioxidant, antidiabetic, antiinflammatory effects	[97,98,99,100]
Melatonin	Hormone	IP3R1	In vitro: Inhibition of OC cell proliferation	Delayed sleep phase syndrome treatment	[101,102]
3,4-dihydroquinazoline derivatives	Experimental compounds	T-type Ca^2+^ channel blocker	In vitro: Alteration of cell-cycle progression, slowing of proliferation, and increased cell death processes	-	[88]
lomerizine	Antimigrainous drug	L and T-type Ca^2+^ channel blocker	In vitro: reduction of stemness and induction of apoptosis in CSCs	Antihistamine and anti-serotonin effects	[103,104]
manidipine, lacidipine, benidipine	Antihypertensive drugs	L-type Ca^2+^ channel blockers	In vitro: reduction of stemness and induction of apoptosis in CSCs	-	[103]
Soricidin, SOR-C13, and SOR-C27	Natural oligopeptide and analogs	TRPV6	In vivo: reduction of growth of OC cell tumor xenografts in mice	-	[105]

These interesting results highlight the need for further studies on the mechanisms through which VGSC, in particular Nav1.5, promotes tumor growth and metastasis.

Concerning ENaC, it has been demonstrated that *SCNN1A*, which encodes the α-subunit of ENaC, is highly expressed in OC samples and cell lines and that OC patients with high *SCNN1A* expression show poor overall survival and progression-free survival [106]. *SCNN1A* knockdown remarkably suppressed SKOV-3 cell proliferation, migration, and invasion, confirming its role in the onset and progression of OC [106]. ENaC is involved in a process called epithelial–mesenchymal transition (EMT), which is a complex developmental program that enables epithelial tumor cells to assume a mesenchymal, fibroblast-like cell phenotype, acquiring highly metastatic property [106]. In EMT, OC cells expressed low levels of the epithelial marker E-cadherin and high levels of mesenchymal markers, such as vimentin, N-cadherin, and Snail [107,108]. SCNN1A silencing led to increased expression of E-cadherin and reduced expression of Vimentin, N-cadherin, and Snail, suggesting that SCNN1A facilitates the EMT and consequently metastasis in OC cells (Figure 1) [106]. These findings highlight the importance of sodium channels in cancer development and pave the way for new therapeutic strategy against OC.

## 3. Potassium Channels

Potassium channels (K^+^ channels) are the largest and most varied group of ion channels, being expressed in both excitable and non-excitable cells. Based on their structure and function, these channels can be classified into four main families: Voltage-gated K^+^ channels (Kv); Ca^2+^– or Na^+^– activated K^+^ channels (KCa, KNa); inwardly rectifying K^+^ channels (Kir); and two-pore domain K^+^ channels (K2P) [109].

Using a variety of K^+^ channel inhibitors on the OC cell line A2780, Zhanping et al. demonstrated that Kv channels are the main K^+^ channels affecting OC cell proliferation and cell cycle progression [110]. Among these, Kv10.1 (Eag) and Kv11.1 (hERG) are significantly upregulated in OC cell lines and tissue, contributing to the hyperpolarization of the cell membrane [111]. Such hyperpolarization may increase Ca^2+^ influx into the cell through transport systems (ion channels and transporters) by increasing the driving force for Ca^2+^ [112,113]. Increased intracellular Ca^2+^ may then facilitate the opening of calcium-dependent K^+^ channels (KCa), the expression of which is stimulated by mitogenic factors, thereby maintaining hyperpolarization and driving force for a sustained Ca^2+^ entry [114,115]. This condition also maintains the driving force for Na^+^ dependent nutrient transport and intracellular acidification through NHE1 [113], driving the cells into a more potentially proliferative state. In addition, we can hypothesize that, in tumor cells over-expressing voltage-gated Ca^2+^ channels (especially T-type channels) and Na^+^ channels, the depolarized resting membrane potential of non-excitable cells may force these channels to enter an inactivated state. Thus, membrane hyperpolarization at rest may remove channel inactivation and allows Ca^2+^ or Na^+^ influx at any further little depolarizing oscillation. Finally, an alternative or complementary hypothesis is that opening of certain K^+^ channels, independently of cell hyperpolarization, may contribute to cell volume regulation, which is critical during cell cycle progression [113].

In this scenario, Eag and HERG channels have been studied as possible molecular targets for anticancer drug design. Indeed, a variety of blockers was shown to inhibit OC cell viability through various pathways. Kv channel inhibition disrupts cell cycle, increasing the proportion of cells in G0/G1 phase with respect to S/G2/M phase (Figure 1). More recently, knockdown of HERG reduced proliferation, migration, and invasive ability of SKOV-3 cells, confirming HERG involvement in the pathogenesis of OC [60,115]. For example, the use of Eag blockers, such as imipramine and clofilium, increased the proportion of SKOV-3 cells undergoing early apoptosis, whereas treatment with the HERG channel specific blockers, E-4031 and ergtoxin, affected the cell cycle, resulting in the block of OC cells in the S phase (Table 1 and Figure 1) [55]. In addition, the natural alkaloid berberine (BBR) induced a strong cytotoxic effect against OC cells through inhibition of hERG channels (Table 1) [60]. Additional studies would be necessary to define the mechanisms by which BBR induces hERG1 channel blocking and by which hERG1 inhibition mediates phenotypic alterations in OC.

An important problem with the use of HERG blockers is the risk of side effects, specifically regarding ventricular arrhythmia and a slowed cardiac repolarization. Maybe the use of state-dependent blockers targeting open Kv11.1 channels in cancer cells, while sparing inactivated cardiac Kv11.1 channels might be helpful [116]. Another strategy to generate highly selective inhibitors of potassium channels is represented by the use of monoclonal antibodies. The first monoclonal antibody against Eag, mAb56, was shown to inhibit OC cell growth in vitro (Table 1) [62], thereby encouraging further studies.

Among the KCa family, the maxi-conductance BK channel KCa1.1 (*KCNMA1* gene) plays an important role in the progression of OC cancer, contributing to cell proliferation and migration [117]. The KCa1.1 channel is over-expressed in several cancers including glioma [118] and hormone-sensitive tumors like breast, prostate and ovarian cancer [119,120]. Its role in OC has been studied by using selective BK channel blockers, such as iberiotoxin and tetrandrine (Table 1) [63]. Tetrandrine inhibits BK channel currents in OC cells and OC cell proliferation by inducing G1 phase arrest and accelerating apoptosis (Figure 1). Inhibition of BK channels also reduced the expression of the heat shock proteins hsp90 and hsp70, which are molecular chaperones involved in the folding and maturation of key regulatory proteins, including p53 [121]. Thus, the decrease of hsp expression may favor the expression of p53, p21, and Bax proteins, thereby triggering apoptosis (Figure 1) [63].

On the other hand, the opening of BK channels by NS1619 inhibits proliferation of the OC cell line A2780 in a time- and dosage-dependent manner (Table 1). The efflux of K^+^ from the cell results in cell shrinkage and the release of cytochrome c and activation of caspase, inducing apoptosis [65]. Thus, activity of BK channels finely tunes OC cell survival (Figure 1).

Recent studies revealed an involvement of KCa2.3 (*KCNN3* gene) and KCa3.1 (*KCNN4* gene) in OC pathogenesis [122,123]. KCNN3 mRNA expression was significantly lower in OC tissues compared with normal controls, suggesting its negative contribution in OC progression [122].

Blockage of the KCa3.1 or its down-regulation in SKOV-3 human OC cells prevented OC migration as a consequence of a loss of interaction between KCa3.1 and the purinergic receptor P2Y2 [123].

An emerging class of K^+^ channels involved in OC is represented by the ATP-sensitive K^+^ channel Kir6.2/SUR2. Such channel results from the combination of the inward rectifier K^+^ channel Kir6.2 (encoded by *KCNJ11*) and the sulfonylurea receptor SUR2 (encoded by *ABCC9*) that is essential for the modulation of channel gating in response to the binding of nucleotides or drugs [124]. Recently, it was reported that Kir6.2 and SUR2 genes are downregulated in OC compared to healthy tissues and that high expression of the SUR2 gene is associated with improved overall survival in all OC patients [67]. The stimulation of the Kir6.2/SUR2 channel activity with the pharmacological activator minoxidil (Table 1) caused a reduction of OC cells proliferation and tumor growth in an OC xenograft model, producing mitochondrial disruption and severe DNA damage [67]. Thus, repurposing of regulatory agency approved K^+^ channel activators could pave the way for novel therapeutic approach in OC, able to improve OC patients’ benefits.

Regarding the K2P channel family, the main members involved in OC are the TWIK-related acid-sensitive potassium channel 3 (TASK-3 encoded by *KCNK9*) and the TWIK- related K^+^ channel type 1 and 2 (TREK-1 encoded by *KCNK2* and TREK-2 encoded by *KCNK10*). Many studies have established *KCNK9* as a proto-oncogene, whose amplification promotes cell proliferation [125]. In keeping with this, an artificial point mutation (G95E) in *KCNK9* abolished TASK-3 channel activity and oncogenic activities, including proliferation, resistance to apoptosis, and tumor cell growth [126]. Pharmacological inhibition of TASK-3 by methanandamide and zinc reduced cell proliferation and apoptosis in the SKOV-3 and OVCAR-3 cell lines (Table 1 and Figure 1). Conversely, increased immunopositivity of TASK-3 in OC patients was associated with clinically significant increased survival, in contrast with the definition of TASK-3 as an oncogene. Therefore, as for BK channels, TASK-3 channels might have a dual role depending on the level of expression/activity, suggesting that results of K^+^ channel modulation may be quite unpredictable [69].

Interestingly, TREK-1-blocking agents, such as curcumin, reduced OC cells proliferation and increased late apoptosis processes, suggesting that these channels could represent a new therapeutic option for OC that requires further investigation (Table 1 and Figure 1) [62].

As for TREK channels, TREK-1 overexpression was found in OC cells and tissues and was associated to a significant increase in cancer cell proliferation. A variety of natural compounds has been studied for their anticancer properties, including curcumin. This polyphenol extracted from the turmeric plants has gained attention for its antioxidant, anti-inflammatory and antimicrobial activities. Curcumin exhibits antioxidant effect through free-radical-scavenging activity and has been involved in many signaling pathways and molecular targets (growth factors, inflammatory cytokines, and apoptotic proteins) that influence cancer growth [127]. Interestingly, it has been demonstrated that curcumin is able to block TREK-1 expression, inducing late apoptosis processes and determining a reduction of OC cells proliferation (Table 1 and Figure 1) [72]. Thus, these results pinpoint TREK-1 channel as a potential target for new therapeutic option for OC.

## 4. Chloride Channels

While most studies have focused on the role of potassium channels up to now, chloride channels have recently gained more attention in carcinogenesis [128]. Like other ion channels, chloride channels and downstream signaling are involved in tumor growth and aggressiveness through the regulation of cell motility, cell cycle progression or apoptosis resistance [129].

Mammalian chloride channels include five classes classified according to their regulation: voltage-gated chloride channels (CLCs), cystic fibrosis transmembrane conductance regulator (CFTR), volume-regulated chloride channels (VRAC), calcium-activated chloride channels (CaCCs), and ligand-gated chloride channels (GABA (γ-aminobutyric acid) and glycine-activated) [130]. Additionally, chloride intracellular channel (CLIC) proteins are the most recent Cl- channels to be discovered and are classified separately from other Cl- channels [131].

Chloride channels are involved in many different cellular functions that include the regulation of the cell excitability, ion homeostasis, trans-epithelial fluid transport, pH level, and cell volume regulation. The latter is particularly relevant for cancer cell migration and infiltration [130]. These channels also contribute to the regulation of the cell cycle, probably as key players in the progression from G1 to S phase [132].

The heterogeneity of chloride channel activity suggests a multitask contribution to OC tumorigenesis. The study of the effects of various inhibitors of CLC and VRAC channels on the OC A2780 cell line demonstrated that treatment with niflumic acid (NFA), tamoxifen and 5-nitro-2-(3-phenylpropylamino)-benzoate (NPPB) inhibited cell proliferation and suppressed cell adhesion and invasion (Figure 1 and Table 1) [79,80]. Further in vitro studies also revealed that ClC-3 silencing by antisense oligonucleotides decreased protein levels of MMP-2, MMP-9, and VEGF in culture medium of SKOV3 cells, confirming ClC-3 involvement in proliferation, invasion, and migration of OC cells (Figure 1) [80].

CFTR is a cyclic AMP-dependent chloride ion-conducting channel expressed mainly in epithelial cells, including the female reproductive tract [86]. Xu et al. investigated for the first time the relationship between CFTR and OC, analyzing CFTR expression in human epithelial ovary cells in normal conditions and in cancer through immunohystochemical staining [133,134]. Their results showed that CFTR expression was significantly increased in OC compared with both benign tumor and normal ovarian tissue and that high expression levels were correlated with poor prognosis, advanced histological grades, and an increased CA125 biomarker level. The role of CFTR was also investigated in vitro in SKOV3 and A2780 cells and in vivo. The knockdown of CFTR suppressed the malignant behavior of OC cells, including cell proliferation, colony formation, migration, invasion, and adhesion (Figure 1). Furthermore, inhibition of CFTR inhibition in vivo suppressed xenograft tumor development, pinpointing CFTR as a potential biomarker in OC [135].

Ca^2+^–activated chloride channels are known to be involved in the regulation of carcinogenesis and are believed to be important emerging drug targets in cancer [136,137]. In particular, a member of the Ca^2+^–activated chloride channels, TMEM16A, also known as ANO1, was proposed to contribute to tumor growth and invasion in head and neck squamous cell carcinomas, breast cancer, hepatocellular carcinoma, lung cancer, gastric cancer, and prostate cancer [138,139,140,141,142,143]. TMEM16A is upregulated also in human epithelial OC cells and peripheral blood mononuclear cells (PBMCs) from patients with OC and its inhibition suppresses growth and invasiveness of OC cells and xenograft tumors in mice (Figure 1) [144]. It is worth noting that TMEM16A gene expression is upregulated in PBMCs from patients with serous OC, but reduced in patients after surgical removal of tumor. All these results suggest that the change of TMEM16A gene expression in PBMCs might be a biomarker for diagnosis or monitoring the progression of OC [144]. However, this hypothesis merits further clinical validation. Since its identification as a CaCC, several regulatory mechanisms of TMEM16A chloride channel have been identified. Among these, it was shown that TMEM16A ion conduction could be affected by lipid environment [145,146]. In cancer cells, lipid distribution in microdomains at the plasma membrane is critical for cancer cell proliferation and migration [147]. Recently, Ye and collaborators revealed that TMEM16A-related chloride currents are involved in the distribution and clustering of phosphatidylinositol 4,5-bisphosphate (PIP2) at the membrane [148]. The cleavage of PIP2 by phospholipase C (PLC) promotes the formation of IP_3_ that, in turn, activates IP_3_R and mobilizes Ca^2+^ from intracellular stores. Thus, disturbing TMEM function or expression may abrogate the formation of such macro-complexes and inhibit the associated downstream signaling pathways [146].

Recent studies have focused attention on TMEM16A channel regulation by the calcium-activated chloride channel regulator 1 (CLCA1) (Figure 1). A growing body of evidence suggests that CaCC-1 may contribute to tumorigenesis in colorectal cancer, pancreatic cancer, and OC [144,149]. Comparative proteomics revealed an increased expression of CaCC-1 in the OC OV-90 cell line and in cell models of tumor aggregate formation (TOV-112D and ES-2). The knockdown of CaCC-1 with the chloride channel blocker NFA or with siRNA reduced the ability of cancer cells to form multicellular aggregates, which is a critical process in metastasis development (Table 1 and Figure 1) [85]. These findings highlight the importance of CaCC-1 in OC progression, and further investigation is warranted to delineate the role of CaCC-1 in the pathogenesis of OC.

An emerging class of chloride channels involved in cancer is represented by the intracellular chloride channels (CLICs) [150]. There are six members in the CLIC family (CLIC1-6), of which CLIC-1, CLIC-3, CLIC-4, and CLIC-5 are the most likely involved in OC. Since CLIC1 and CLIC4 were the first CLIC proteins to be cloned and functionally studied [151], they remain so far the most characterized within the family. These proteins were initially identified as blood biomarkers of OC because human CLIC1 and CLIC4 were found into the blood of xenografted tumor-bearing mice and were significantly elevated in sera of EOC patients [151,152,153,154]. Furthermore, in vitro experiments demonstrated that CLIC1 and CLIC4 knockdown slowed the proliferation and migration of OC cells, indicating a potential role in tumor progression (Figure 1) [155,156]. Singha and collaborators showed that elevated CLIC4 expression, but not CLIC1 expression, was a negative indicator of patient survival, suggesting its potential as a diagnostic and prognostic marker for OC and as a target for therapy [156]. These results have been recently supported by an in silico analysis that correlated the expression of CLIC proteins with cancer related patient mortality. The data showed that low or high CLIC1 expression in the OCs does not influence patient survival, but that high CLIC3 and CLIC4 expression correlates with poor patient survival in all the six cancers analyzed, including OC [157]. It is worth noting that, although CLIC proteins can form functional chloride channel in lipid bilayer, their actual function in live cells remains elusive [158]. Thus, if their involvement in cancer is documented, there is no available data showing that they act effectively as chloride channels.

## 5. Calcium Channels

Calcium is an essential signal transduction element involved in many different cell processes such as proliferation, differentiation, growth, cell death, and apoptosis [159]. Calcium fluxes through cytoplasmic and intracellular Ca^2+^ channels are required for cell cycle progression from G1/S phase through mitosis. Conversely, depletion of intracellular calcium blocks the cell cycle in the G0/G1 and S phases [160]. Thus, the remodeling of Ca^2+^ homeostasis is thought of as a critical hallmark of cancer cells. In OC, the calcium channels involved in calcium signaling pathways promoting cancer behaviors are mostly voltage-gated calcium channels (VGCC), non-voltage activated calcium channels (belonging to the TRP superfamily, see Section 6), and intracellular calcium channels such as the IP_3_R and Orai families. Among VGCC, T-type Ca^2+^ channels resulted the most studied for its altered expression in several cancers, including OC. Expression of T-type Ca^2+^ channel is greatly increased in OC A2780 and H08910 cell lines compared with their benign counterparts [87,88]. Treatment of these cells with T-type Ca^2+^ channel blockers, such as mibefradil or 3,4-dihydroquinazoline derivatives (e.g., KYS05090), disturbs cell-cycle progression, slows proliferation, and enhances cell death (Figure 1 and Table 1). The mechanism for the induction of those effects involves caspase dependent apoptosis accompanied by a reduction of the antiapoptotic protein, survivin [89]. Survivin is a direct downstream target of the PI3K/AKT pathway: the activation of this pathway induces the inactivation of FOXO-containing transcription repressor complexes and increases expression of survivin. Inhibition of T-type Ca^2+^ channels decreases AKT phosphorylation and modifies FOXO expression, which culminates in reduced survivin expression (Figure 1) [89]. Although the direct molecular connection between T-type channels and the PI3K/AKT/FOXO pathway is still unknown, inhibitors of T-type channels or survivin expression could be developed. Aberrant expression of VGCC in OC was also implicated in OC cell migration. The Ca^2+^ influx mediated by L-type calcium channels was responsible for the activation of the MAPK signaling cascade and ERK signaling pathway, which subsequently led to an increase of OC cells migration [161]. Growing evidence also revealed a tight cross-talk between L-type Ca^2+^ channel and lipid metabolism in OC progression [162]. High level of lysophosphatidic acid (LPA) and arachidonic acid (AA)-derived eicosanoids were found in ascites of OC patients and in vivo studies referred to them as triggers of the Ca^2+^ influx in the OC cells inducing proliferation, migration or drug resistance [163,164]. The ability of LPA to increase cellular Ca^2+^ entry could be due to a variety of signaling pathways. It mainly acts via GPCRs, activating Gq protein and increasing the cytosolic Ca^2+^ concentration through the classic phospholipase C (PLC)-dependent pathway. Recent studies also hypothesized that LPA-induced Ca^2+^ mobilization may be due to a directly modulation by LPA of L-type Ca^2+^ channels and Ca^2+^ activated K^+^ channels [162]. (Indeed, treatment of SKOV-3 OC cells with the L-type Ca^2+^ channel blocker nifedipine (Table 1) significantly blocked LPA-induced cell migration and adhesion in OC cancer [93].

In addition, AA may exert a critical role in regulating cytosolic Ca^2+^ concentration and consequently cell migration and invasion. It was demonstrated that AA directly inhibits TRPC3 (See Section 6) in mammary MCF-7 cells, attenuating breast cancer cell proliferation and migration [165]. However, the link between lipid mediators and Ca^2+^ channels needs to be further investigated in OC in order to suggest novel therapeutic strategies for OC treatment.

Calcium cytosolic concentration is also regulated by intracellular channels allowing Ca^2+^ movement between subcellular compartments. Over the last years, a critical role of the IP3Rs and Orai1-STIM1 in OC progression has become increasingly apparent in numerous studies. IP3Rs are Ca^2+^ channels, mainly located in the endoplasmic reticulum (ER), responsible for Ca^2+^ release from ER into the cytosol after activation by IP3 and modulation by calcium [166]. The close proximity of the ER to mitochondria, lysosomes, and nucleus, has allowed IP3Rs to emerge as critical regulators of cell fate. Three IP3R homologous isoforms, IP3R1s, IP3R2s, and IP3R3s, have been identified as key sites for regulation by pro- and anti-apoptotic factors. The flow of Ca^2+^ specifically from IP3Rs caused mitochondrial permeability transition, thereby releasing several pro-apoptotic factors into the cytoplasm, which activates the apoptotic cascade. The role of IP3R1 isoform in apoptosis induction was widely described in a variety of cancer cells. In OC, it was demonstrated that natural compounds like sulforaphane (SFN) (Table 1) [97] or hormones like melatonin (Table 1) [101] may act as potential chemotherapeutic agents in OC cells lines, partially upregulating IP3R1. In keeping with this, in vivo experiments showed that SNF treatment was able to reduce OC volume in athymic nude mice with OC-induced by A2780 cells injection. When SFN was co-administered with IP3R blocker, tumor volume was higher compared to sole SFN treatment, but still lower than in untreated mice. These results supported the idea that IP3R1 was involved in pro-apoptotic processes, taking part to the tumor suppressor effect in OC [97]. However, the expression profile of IP3R1 remains uncertain in cancer progression because other results showed contrasting actions of this channel in tumor progression and drug resistance. For instance, an enhanced activity of IP3R1 in prostate cancer promotes cell survival and resistance to hormonal deprivation therapy [167]. More recently, it was reported that IP3R1 plays a different role in MDA-MB-231 breast cancer cells compared to JIMT1 breast cancer cells, DLD1 colorectal and A2780 OC cells [94]. Treatment of these cell lines with the VGCC blocker nifedipine (Table 1) induced an increase of cytosolic Ca^2+^ levels in A2780 and DLD1 cells, whereas no changes have been seen in MDA-MB-231 and JIMT1 breast cancer cells. The high cytoplasm Ca^2+^ concentration in A2780 and DLD1 cells was mainly due to the upregulation of Cav1.3 and IP3R1, which allows linking to the activation of apoptotic process. Indeed, IP3R1 silencing determined an increase in proliferation and migration in DLD1 cells, whereas a decrease of these processes have been seen when IP3R1 was silenced in MDA-MB-231. These results support different functions of the IP3R1 in the invasiveness of various cancer cells in response to nifedipine treatment [168]. In stark contrast to IP3R1, it was demonstrated that IP3R3 has anti-apoptotic and proliferative function in several cancers, including OC [168]. IP3R3 expression was upregulated in glioblastoma, colorectal carcinoma, and breast cancer, and was correlated to an enhanced capacity of cancer cell migration and invasion [168]. Indeed, the inhibition or downregulation of IP3R3 also abrogated proliferative and invasive phenotype and extended survival rate [169,170].

The Ca^2+^ release from intracellular stores determined the activation of Store Operated Ca^2+^ Entry (SOCE), a process by which extracellular Ca^2+^ entries into the cytoplasm to refill the internal Ca^2+^ stores. This event is controlled at a molecular level by the canonical TRP channels, the Ca^2+^ release-activated calcium channel protein 1 (ORAI1) and the ER Ca^2+^ sensors STIM1 (stromal interaction molecule 1) and STIM2 [171]. A number of studies have shown that the overexpression of these channels is positively correlated to cell proliferation, migration, and invasion in several cancers, including glioblastoma, cervical cancer, and breast cancer [172,173]. Recently, it was found a tight connection between Orai1-STIM1 expression and placental growth factor (PlGF) in OC cells. Exposure of OC cells to PlGF triggered a significant increase of Orai1 as well as STIM1 transcript and protein levels, enhancing SOCE and the expression of Hypoxia Inducible Factor HIF1α, which may support proliferation and survival of metastatic tumor cells [174]. Thus, targeting all these pathways may offer new therapeutic options for the treatment of OC.

## 6. TRP Channels

TRP channels are calcium-permeable and non-selective ion channels grouped into six subfamilies, including TRPC (“C” for canonical), TRPV (“V” for vanilloid), TRPM (“M” for melastatin), TRPA (“A” for ankyrin), TRPP (“P” for polycystic), and TRPML (“ML” for mucolipin) [175]. TRP subfamilies could be involved in calcium influx and downstream pathways, regulating cell survival, proliferation, and apoptosis. Upregulation or downregulation of TRP channels is linked to various phases of tumor progression, and strongly correlates with clinical parameters, such as overall survival and progression-free survival.

Immunohistochemistry of healthy and OC tissue revealed that TRPV1 and TRPV6 channels were markedly increased in OC tissues compared to healthy tissues [176,177]. TRPV6 mRNA and protein are also highly expressed in all OC subtypes, both in early and in late stage, indicating TRPV6 mRNA levels as a promising biomarker for the presence of OC [178]. In prostate and breast cancers, elevated TRPV6 and the resulting sustained elevation of intracellular Ca^2+^ promote the activation of Nuclear Factor of Activated T-cell transcription factor (NFAT), which activates genes involved in cell proliferation and migration [179,180]. Thus, reduction of TRPV6 activity by decreasing its expression or by pharmacological intervention may represent a way to develop new therapeutic molecular interventions in cancer. Indeed, targeting TRPV6 with specific channel blockers (such as soricidin and its derivates SOR-C13 and SOR-C27) reduces growth of OC SKOV-3 tumor xenografts in mice, further supporting this channel as a viable target (Figure 1 and Table 1) [178]. Moreover, the peptide inhibitor of TRPV6, SOR-C13, completed a phase 1 clinical trial [181], resulting as safe and well tolerated in patients with advanced epithelial OC, without evidence of hematological, neurological or other significant toxicities.

Evidences on the role of TRPC channels in cancer are expanded with their detection in a number of different tumors including breast, ovary, colon, thyroid, and pancreas [182,183,184,185,186]. Several spliced variants of TRPC1, TRPC3, TRPC4, and TRPC6 were found in OC [187]. TRPC3 channels resulted overexpressed both in human OC tissue and in OC cells, suggesting a role in OC progression. Accordingly, knockdown of these channels reduced the proliferation of OC cells and suppressed tumor formation in mice after OC cells injection, suggesting a functional role for TRP-mediated Ca^2+^ entry in cell proliferation and invasion [105]. Recently, it was also demonstrated that TRPC3 expression is upregulated in OC cells after estrogen stimulation, promoting proliferation and migration of cells through intracellular calcium signaling pathways [188].

Lastly, TRPM7 is highly expressed in OC cells and tissues and its expression level is significantly associated with poor prognosis in patients with OC [189]. Silencing of TRPM7 with siRNA inhibited OC cells proliferation, migration, and invasion, through direct regulation of calcium influx that, in turn, modulates many signaling transduction pathways including Akt, Src, and p38 (Figure 1) [190,191].

## 7. Ion Channels and Drug Resistance

The high mortality rate in OC is related to late diagnosis in advanced clinical stages, metastasis in the peritoneal cavity, and resistance to chemotherapy [192]. It is estimated that 75% of patients who are highly responsive to initial therapy, relapse within 2 years because of acquired resistance [193]. For instance, resistance to platinum agents remains a significant obstacle to overcome. Various mechanisms causing cell drug resistance have been described in vitro and in vivo.

**Figure 2:**
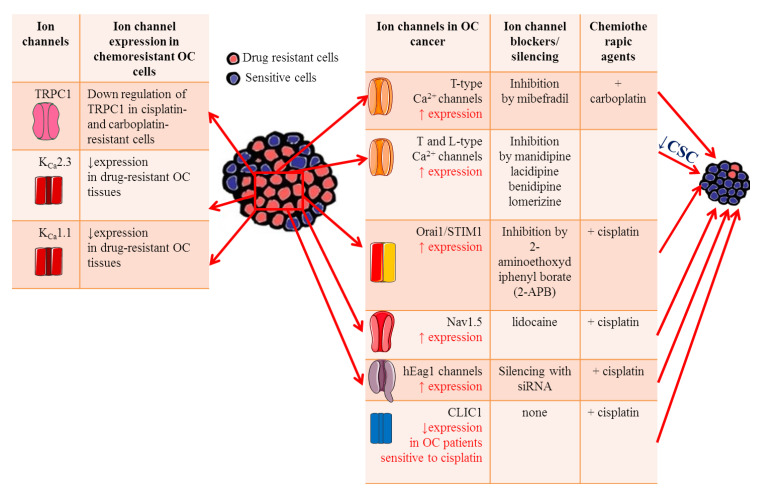
Ion channels roles in OC drug resistance. Table on the left summarizes the main ion channels likely involved in OC drug resistance, focusing on their expression level on OC cells and tissues. Table on the right lists the effects of ion channel blockers on chemosensitivity to platinum-based agents.

Ion channels have been shown to play key roles in chemosensitivity and several ion channels have been shown to modify sensitivity to neoplastic drugs by modulating apoptosis pathways or the efficiency of drug permeation due to electrochemical gradients. The abnormal expression of antiapoptotic proteins is often associated with OC aggressiveness and resistance to cytotoxic therapy [194], suggesting that intervention on this pathway may be an effective approach to increase OC response to therapy. In this respect, inhibition of T-type Ca^2+^ channels with the antagonist mibefradil or siRNA induces proapoptotic responses through reduced expression of the antiapoptotic gene *survivin* (Table 1) [161]. Thus, the inhibition of *survivin* expression enhances the ability of platinum agents to increase DNA double strand breaks and induce apoptosis in OC [195]. Indeed, combining carboplatin with mibefradil synergistically increases apoptosis and inhibits growth of platinum-resistant OC cells in vitro. More importantly, in vivo pretreatment with mibefradil in carboplatin-based therapy reduced xenograft tumor burden in mice model of platinum-resistant OC (Figure 2) [161], supporting the idea that T-type Ca^2+^ channel blockers could be useful to improve patients’ response to platinum agents. Few studies focused on the roles of Orai1 and STIM1 in OC drug resistance. One work from Schmidt and collaborators showed that these channels are highly expressed in the A2780 cisplatin-resistant OC cells compared to A2780 non-resistant cells, leading to elevated SOCE [196]. Pharmacological inhibition of Orai1 with 2-aminoethoxydiphenyl borate (2-APB) increased cisplatin-induced apoptosis of A2780 cisplatin-resistant OC cells and abrogated the differences in cisplatin sensitivity between them and A2780 non-resistant cells. Taken together, these data suggest that enhanced SOCE could contribute to drug resistance in OC cells and might become potential targets in OC treatment [195]. The role of TRPC1 channels in platinum resistance has also been investigated. A marked decrease in TRPC1 mRNA levels was measured in cisplatin and carboplatin-resistant OC cells compared with their sensitive counterparts [197], suggesting a TRPC1 contribution to drug resistance (Figure 2). A mechanism that contributes to cisplatin resistance in OC is autophagy. A tight interaction was found between TRPC1 and two proteins involved in ERK-mediated autophagy (PIK3C3 and SPARCL1) [198,199], confirming the role of TRPC1 in the regulation of autophagy and in drug resistance in OC. More recently, it was demonstrated that Nav1.5 was involved in cisplatin resistance in OC. In vitro experiments showed that lidocaine enhanced OC cells sensitivity to cisplatin by blocking Nav1.5 and FAK/Paxillin signaling pathway. The OC cells treated with cisplatin and lidocaine combination expressed higher levels of apoptotic proteins such as caspase-3 and 8, and lower level of the antiapoptotic protein Bcl-2 [48]. Thus, targeting these channels maybe a promising strategy for overcoming cisplatin resistance.

Drug resistance phenomenon in OC is reportedly attributed to the existence of cancer stem cells (CSC), which establish a reservoir of cells with the exclusive capacity of self-renew. These cells are generally resistant to conventional anticancer treatments [200]. Thus, the discovery of drugs that selectively target CSCs may help preventing cancer recurrence, improving the survival rate of OC patients. Indeed, Lee and collaborators screened an FDA-approved compound library of ~1000 compounds on OC stem cells (CSCs) and found that four calcium channel blockers (manidipine, lacidipine, benidipine, and lomerizine) had anticancer effects against ovarian CSC by reducing stemness and inducing apoptosis in CSCs (Figure 2 and Table 1) [103]. These results suggest that calcium channels may represent a selective target for new therapeutic approaches in the prevention of OC recurrence.

The importance of K^+^ influx in cisplatin resistance in OC was showed for the first time by Sharp et al., who demonstrated that the K^+^ ionophore amphotericin B could sensitize cells to cisplatin and other platinum-containing agents (Table 1) [76]. Reduction of intracellular K^+^ concentration by amphotericin B or the Na-K-Cl cotransporter inhibitor bumetanide boost cisplatin-induced apoptosis (Table 1) [78,201]. These early results suggested that there might be a tight correlation between the expression levels of K^+^ channels and the sensitivity to cytotoxic drugs. Accordingly, hEag1 channel silencing increased the chemosensitivity of SKOV3 cell lines to cisplatin, promoting apoptosis through the NF-κB pathway (Figure 2) [202]. The decreased hEag1 expression found in OC tissue of patients treated with cisplatin-based adjuvant chemotherapy was correlated with a favorable prognosis and predicted higher sensitivity to cisplatin treatment.

The *KCNN3* gene encoding the calcium-activated K^+^ channel KCa2.3 was predicted to be one of 1298 genes contributing to drug resistance in OC [203]. Using immunohistochemistry and big data analyses, Liu and collaborators found a significantly lower expression of *KCNN3* in drug-resistant OC tissues, confirming an involvement of KCa2.3 channels in drug resistance (Figure 2) [114,122]. It was further hypothesized that KCa2.3 and TRPC1 may act in concert in drug resistance through inhibition of drug-induced cell death [204]. This promising combination strategy warrants further study. In addition, the decreased *KCNMA1* expression in OC cell lines was associated with minor sensitivity to platinum-based chemotherapy (Figure 2) [205].

Regarding chloride channels, OC patients with lower CLIC1 levels showed increased sensitivity to cisplatin treatment [154], likely because CLIC1 participates in the regulation of anti-apoptosis signaling pathways in response to chemotherapy (Figure 2).

Altogether, these results shed new light on how ion channels contribute to chemotherapy resistance, providing a potential therapeutic alternative for OC patients who frequently succumb to platinum resistant disease.

## 8. Concluding Remarks and Perspectives

A variety of ion channels are remarkably expressed in human OC cells and tissues, where they are involved in the development of various malignant behaviors, including proliferation, migration, invasion, and apoptosis resistance. For some of these channels, a correlation of their expression levels with prognosis or resistance to cytotoxic drugs has also been reported, supporting a putative role as predictive markers for combination chemotherapy regimens. In line with this, the specific inhibition of some ion channels increases the cytotoxicity induced by different chemotherapeutic drugs, thus opposing tumor resistance. Accumulating data have revealed that the aberrant expression of ion channels in OC greatly contribute to the main hallmarks of OC process, from initial proliferation to metastasis to chemoresistance. The T-type Ca^2+^ channels, for example, are overexpressed in ovarian cancer where they are involved in tumor growth and its chemoresistance to platinum-based drugs [89]. Indeed, blocking T-type Ca^2+^ channels with mibefradil during administration of carboplatin sensitizes platinum-resistant ovarian tumors to the anticancer drug-mediated toxicity [206]. Most studies reported in this review describe the implication of one channel in the regulation of OC development and progression. However, it is likely that channels may work in tandem or in more complex network to promote specific cell function. Studies about complexes of ion channels are emerging to explain their involvement in the control of these processes. For example, voltage-gated Na^+^ channels can be associated with NCX and NHE1 cotransports to stimulate tumor cell metabolism and promote cancer cell invasion and metastatic progression [35]. In colorectal cancer cells, the simultaneous activation of KCa3.1 and inhibition of Kv11.1 with riluzole synergistically reduces tumor proliferation while increasing apoptosis and overcoming cisplatin resistance [207]. Thus, the identification of the complete set of ion channels involved in OC and their interrelationships might allow to set up new promising therapeutic approaches, pointing for instance to a combination of different channel inhibitors or employing the drugs together with chemotherapeutics. Furthermore, the use of selective modulators directed on transmembrane ion channels allows reaching easily the target without entering the cells. This means that these drugs are not subjected to efflux pumps, bypassing this major chemoresistance mechanism. Membrane localization is also an invaluable opportunity for the use of selective monoclonal antibodies [208].

Importantly, many of the available pharmacological modulators of ion channels are already successfully used for the treatment of various non-oncological diseases, which may facilitate their repurposing in OC treatment. The use of clinically approved drugs could bring many benefits including known safety profile and well understood pharmacokinetics in humans, as well as saving time and money in drug development.

However, there is a risk that these modulators may have off-target effects on normal physiological function, because many of the channels identified in OC cells are also expressed in healthy normal cells. In this regard, it would be important to focus on and selectively target those ion channels having aberrant overexpression in the tumor tissues compared to the normal counterpart tissue or showing cancer-specific isoforms/splice variants. Antibodies or small molecules that target tumor-specific isoforms may represent a way to increase the sensitivity and specificity of ion channel-targeted therapies in cancer. An example is the highly selective monoclonal antibody against the oncogenic Kv 10.1 potassium channel, which has been demonstrated to inhibit tumor growth both in xenograft models of human melanoma cells and in xenografted human pancreatic cancer [209]. Antibodies or fragments against ion channels might be also useful for cancer diagnosis [210]. However, the monoclonal antibody-based approach is limited by the continuous dynamics of the tumor environment and by the size-dependent tissue penetration, and further investigation is needed to overcome these limitations. In addition, it is worth noting that the activity and expression of ion channels may vary in a cell cycle-dependent manner and between tumor cell subtypes (e.g., cancer stem cells versus parenchymal cells), limiting the efficacy of ion channel modulators.

The study of ion channels expression in OC has been steadily gaining interest with time, also because of their involvement in cancer drug resistance. Several benefits could be obtained through a combined strategy of ion channel-targeting drugs and chemotherapy, improving pharmacological response in chemoresistant OC patients. Further studies are warranted in animal models and in humans, in order to develop new specific therapeutic approaches.
